# Supplementary material for: A Critical View of the Application of the APEX Software (Aqueous Photochemistry of Environmentally-Occurring Xenobiotics) to Predict Photoreaction Kinetics in Surface Freshwaters
Source: Molecules. 2019 Dec 18;25(1):9. doi: 10.3390/molecules25010009 (PMC7017383; doi:10.3390/molecules25010009)
Supplement: Supplementary file 1 [file molecules-25-00009-s001.zip › 3.2.4_gcc-4.4.0.zip!/3.2.4_gcc-4.4.0/3.2.4_gcc-4.4.0/doc/octave/html/interpreter/Techniques-used-for-Linear-Algebra.html]

Techniques used for Linear Algebra - Untitled


Next: Basic Matrix Functions,
Up: Linear Algebra


---

### 18.1 Techniques used for Linear Algebra

Octave includes a polymorphic solver, that selects an appropriate
matrix factorization depending on the properties of the matrix itself.
Generally, the cost of determining the matrix type is small relative to
the cost of factorizing the matrix itself, but in any case the matrix
type is cached once it is calculated, so that it is not re-determined
each time it is used in a linear equation.

The selection tree for how the linear equation is solve or a matrix
inverse is form is given by

1. If the matrix is upper or lower triangular sparse a forward or
   backward substitution using the lapack xTRTRS function, and goto 4.


   - If the matrix is square, hermitian with a real positive diagonal,
     attempt Cholesky factorization using the lapack xPOTRF function.- If the Cholesky factorization failed or the matrix is not
       hermitian with a real positive diagonal, and the matrix is square, factorize
       using the lapack xGETRF function.- If the matrix is not square, or any of the previous solvers flags
         a singular or near singular matrix, find a least squares solution using
         the lapack xGELSD function.

The user can force the type of the matrix with the `matrix_type`
function. This overcomes the cost of discovering the type of the matrix.
However, it should be noted that identifying the type of the matrix incorrectly
will lead to unpredictable results, and so `matrix_type` should be
used with care.

It should be noted that the test for whether a matrix is a candidate for
Cholesky factorization, performed above and by the `matrix_type`
function, does not give a certainty that the matrix is
Hermitian. However, the attempt to factorize the matrix will quickly
flag a non-Hermitian matrix.
